# "Shping 2" different cellular localizations - a potential new player in aging processes

**DOI:** 10.18632/aging.100063

**Published:** 2009-06-29

**Authors:** Sascha Jakob, Joachim Altschmied, Judith Haendeler

**Affiliations:** Department of Molecular Cell & Aging Research, IUF at the University of Duesseldorf gGmbH, 40225 Duesseldorf, Germany; ^1^ Equally contributed senior authors

**Keywords:** Shp-2, nucleus, mitochondria, Telomerase Reverse Transcriptase

## Abstract

The functions of the ubiquitously expressed protein tyrosine phosphatase Shp-2 are dependent on its localization. Cytosolic Shp-2 is known to modulate different pathways involved in cell growth, cell development, tissue inflammation and cellular chemotaxis. But Shp-2 is also localized in the nucleus and the mitochondria. Nuclear Shp-2 forms a complex with the signal transducer and activator of transcription 5 (STAT5) which then binds to DNA and regulates transcription of milk genes. In contrast, nuclear Shp-2 dephosphorylates STAT1 and thereby inhibits gene transcription. In addition, it counteracts the oxidative stress dependent nuclear export of Telomerase Reverse Transcriptase (TERT) mediated by members of the Src kinase family, a process leading to replicative senescence. For the recently found mitochondrial Shp-2 an involvement in the regulation of the cellular redox balance is discussed. Shp-2 shows the ability to regulate reactive oxygen species formation in the mitochondria. There are hints that mitochondrial Shp-2 and Src are involved in the regulation of respiratory chain activity. Since a substantial fraction of TERT has been found in the mitochondria, it is hypothesized that mitochondrial Shp-2 acts as a positive regulator of TERT in the mitochondria, similar to its nuclear role. Taken together, Shp-2 seems to be a new player in aging processes.

Shp-2 is a ubiquitously expressed protein tyrosine phosphatase, which contains two N-terminal Src homology 2 (SH2) domains and a C-terminal protein tyrosine phosphatase domain. Several years of research established an important role for cytosolic Shp-2. It is known to modulate different pathways involved in cell growth, cell development, tissue inflammation and cellular chemotaxis due to its well described function to dephosphorylate receptor tyrosine kinases (reviewed in [1]). However, over the last years it has become clear that Shp-2 is also localized in the nucleus and in the mitochondria where it exerts different functions.

In 2002 Chughtai et al reported a nuclear localization of Shp-2 associated with the signal transducer and activator of transcription 5 (STAT5). The stimulation of mammary cells with prolactin induced the nuclear translocation of Shp-2 in a complex with STAT5. Formation of this complex and tyrosine phosphorylation of STAT5 in response to prolactin requires the SH2 domain closer to the C-terminus and the catalytic activity of Shp-2. The authors speculated that the nuclear Shp-2/STAT5 complex binds to DNA and regulates transcription of milk protein genes [2], demonstrating a transcriptional regulation by nuclear Shp-2. This provided for the first time evidence for a function of Shp-2 besides dephosphorylation. In contrast, it has been demonstrated that Shp-2 dephosphorylates STAT1 at tyrosine and serine residues in the nucleus and thereby inhibits its transcriptional activity [3]. One may speculate that depending on the mode of its action Shp-2 differently regulates specific STAT proteins. Just recently, we discovered that nuclear Shp-2 seems to be involved in aging processes. Previous findings from our group demonstrated that the enzyme Telomerase Reverse Transcriptase (TERT), which is important for maintaining telomere length and known to delay aging processes, when overexpressed, is tyrosine phosphorylated by Src kinases in the nucleus under conditions of oxidative stress in several cell types, including endothelial cells [4,5]. This tyrosine phosphorylation triggers nuclear export of TERT. Taking into account that cytosolic Shp-2 and the cytosolic Src kinase family can regulate and antagonize each other under certain conditions, we hypothesized that a nuclear Shp-2 also exists in endothelial cells and that this may counteract the Src kinase dependent nuclear export of TERT. Indeed, ablation of endogenous Shp-2 results in increased tyrosine phosphorylation of nuclear TERT and a reduction of telomerase activity in the nucleus. Moreover, overexpression of Shp-2 inhibited oxidative stress induced tyrosine phosphorylation and export of TERT from the nucleus. It has to be noted that this process requires the catalytic activity of Shp-2, since the catalytically inactive mutant Shp-2(C459S) can not pre- vent nuclear export of TERT. Interestingly, overexpression of Shp-2(C459S) reduced nuclear telomerase activity already under basal conditions. This effect was dependent on tyrosine 707 in TERT [6]. One possible explanation for the nuclear export of TERT induced by Shp-2(C459S) under basal conditions could be the significant increase in reactive oxygen species (ROS), which are known to activate the Src kinase family. Indeed, ROS formation is enhanced upon overexpression of Shp-2(C459S) in endothelial cells (Figure 1). These data point to a regulatory role of Shp-2 in the redox balance of cells.

**Figure 1.**
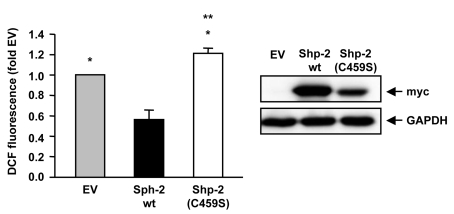
Shp-2 reduces endogenous ROS formation. Endothelial cells were transfected with empty vector (EV), Shp-2 wt or Shp-2(C459S) and endogenous ROS formation was measured using FACS analysis. *p<0.05 versus Shp-2 wt. **p<0.05 versus EV. Data are means +/- SEM (n=6). The Western blot on the right demonstrates expression of Shp-2 wt and Shp-2(C459S), which were detected with anti-myc antibody.

ROS are important signalling molecules for cellular signal transduction. An imbalance of the redox status with a reduced antioxidative capacity and an increased ROS production has been described to play an important role in aging processes as well as in several diseases. Increased ROS can directly damage DNA, proteins and membrane lipids. This leads among others to damage of the electron transport chain, which results in an increased formation of ROS which in turn cause further damage to DNA, proteins and lipids. This vicious cycle seems to play an important role in aging processes and age-related diseases (for review see [7]).

**Figure 2.**
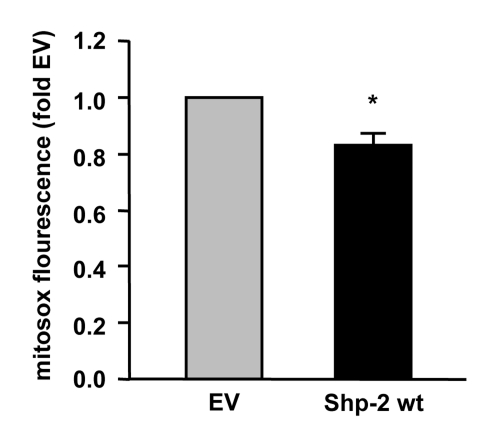
Shp-2 reduces endogenous mitochondrial ROS formation. Endothelial cells were transfected with empty vector (EV) and Shp-2 wt. Mitochondrial ROS formation was measured using mitosox and FACS analysis. *p<0.05 versus EV. Data are means +/- SEM (n=3).

**Figure 3.**
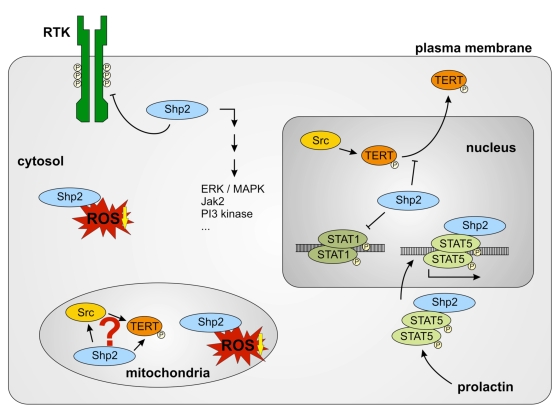
Different functions of Shp-2 in different cell compartments. Cytosolic Shp-2 modulates different pathways by dephosphorylation of receptor tyrosine kinases (RTK). It also decreases cytosolic ROS levels. Nuclear Shp-2 inhibits ROS induced nuclear export of TERT and DNA-binding of STAT1 dimers by dephosphorylation. Prolactin induces the association of Shp-2 and STAT5 and nuclear import of this complex. Shp-2/STAT5 complex binds to DNA and induces transcription of milk genes. Functions of mitochondrial Shp-2 remain unclear. A connection between mitochondrial Src, Shp-2 and TERT may exist. Reduction of mitochondrial ROS formation seems to depend on Shp-2.

Therefore, controlling ROS formation seems to be an interesting tool in delaying aging processes. It is tempting to speculate that nuclear Shp-2 plays an important role in nuclear based aging processes by reducing export of TERT from the nucleus and by reducing ROS formation (figure 1). However, we have also new hints, that Shp-2 may affect mitochondrial ROS production and thus, aging processes which depend on reduced mitochondrial function. New data from our laboratory demonstrate that overexpression of Shp-2 decreases not only ROS production in the cytosol (Figure 1) but also in the mitochondria (Figure 2). Moreover, preliminary results suggest that ablation of Shp-2 increases mitochondrial ROS levels. To specifically measure mitochondrial ROS levels, we used mitosox, a redox-sensitive dye, which first has to enter mitochondria before it can react with ROS. One can speculate, that the observed reduction of mitochondrially derived ROS is connected to a localization of Shp-2 in the mitochondria. Indeed, Salvi et al detected a tyrosine phosphatase activity in the mitochondria of rat brains and identified the responsible phosphatase as Shp-2 [8]. Recently, Arachiche et al showed also the mitochondrial localization of Shp-2 and of the tyrosine kinase Src, which is regulated by Shp-2 [9]. They demonstrated that the complexes of the respiratory chain are substrates of Src, which indicates that respiratory chain activity is partially dependent on tyrosine phosphorylation. Since Shp-2 is an important regulator of Src, Shp-2 is possibly involved in regulation of mitochondrial activity. In line with these findings, we recently demonstrated that TERT is localized in the mitochondria and importantly contributes to respiratory chain activity [10]. TERT deficient mice derived from heterozygous breeding pairs, which show no reduction in telomere length and thus no premature aging phenotype, demonstrated reduced respiratory chain activity in the heart, suggesting an important role for TERT in respiration in vivo [10]. Given the facts that Src kinase family members as well as Shp-2 show mitochondrial localization [9], it is tempting to speculate that similar to nuclear TERT also mitochondrial TERT is positively regulated by Shp-2 in these organelles. This could implicate that mitochondrial Shp-2 in concert with TERT accounts for an intact respiratory chain activity and for reduced mitochondrial ROS formation. Therefore, mitochondrial Shp-2 and TERT could break the above mentioned vicious cycle and thereby may delay aging processes, which depend on mitochondrial dysfunction.

In summary, Shp-2 has the potential to be a yet unknown new important key player in aging processes. Its regulatory function seems to be dependent on its localization within the cell (Figure 3). Nuclear localized Shp-2 counteracts replicative senescence induced by nuclear TERT export and mitochondrial Shp-2 could delay aging processes induced by elevated ROS levels. Therefore, Shp-2 could be an important target for the therapy of diseases connected to aging processes. However, therapeutic interventions aimed at the activation of Shp-2 should take into account the compartment specific functions of this protein.
